# Association between oral health behavior and chronic diseases among middle-aged and older adults in Beijing, China

**DOI:** 10.1186/s12903-023-02764-y

**Published:** 2023-02-14

**Authors:** Dan Guo, Zhenyu Shi, Yanan Luo, Ruoxi Ding, Ping He

**Affiliations:** 1 School of Public Health, Peking University, Beijing, 100191 China; 2 China Center for Health Development Studies, Peking University, Beijing, 100191 China; 3 Department of Global Health, School of Public Health, Peking University, Beijing, 100191 China

**Keywords:** Oral health behavior, Chronic diseases, Middle-aged and older adults

## Abstract

**Objectives:**

To evaluate the association between oral health behavior and multiple chronic diseases among middle-aged and older adults.

**Methods:**

We obtained data of the Beijing Health Service Survey and used multivariate logistic models to estimate the association between oral hygiene behavior and the risk of chronic diseases.

**Results:**

The risk of any chronic diseases (OR = 1.27, 95% CI: 1.18–1.37), cardiovascular diseases (CVD, OR = 1.30, 95% CI: 1.21–1.39), and endocrine or nutritional metabolic disorders (OR = 1.11, 95% CI: 1.01–1.22) was higher in those who with poor oral health behavior. There was no significant correlation between oral health behavior and the risk of diseases of the musculoskeletal, respiratory, digestive, and genitourinary systems.

**Conclusions:**

Poor oral hygiene practices were associated with higher risk of chronic diseases, CVD and diabetes mellitus (DM) among middle-aged and older adults. These findings motivate further studies to evaluate whether improved oral health behavior may prevent the incidence of chronic diseases.

**Supplementary Information:**

The online version contains supplementary material available at 10.1186/s12903-023-02764-y.

## Introduction

The epidemic of chronic diseases poses devastating health consequences for individuals, families, and societies. Globally, 41 million people die from chronic diseases each year, which corresponds to around 71% of all deaths [1]. A significant rise in the prevalence and mortality of chronic diseases has been observed in China as a result of the aging population as well as increased exposure to main risk factors [2]. In 2019, chronic diseases accounted for 88.5% of all deaths in China [3]. Previous studies have found that poor health behaviors, including smoking, physical inactivity, unhealthy diets, and never or rarely brushed tooth, were associated with a higher risk of chronic diseases [4, 5].

Poor oral health is an important public health problem affecting middle-aged and older adults [6]. Specifically, tooth brushing is a simple and low-cost care for maintaining oral health, and it is one of the public health actions recommended by both national and international organizations [7]. Good oral health behavior is very important for effective removal of dental plaque and prevention of periodontal diseases [8]. Caries and periodontal conditions can affect several systemic disorders in addition to their local effects on the dentition and tissues that support the teeth [9]. Previous studies have demonstrated that oral bacteria can reach the bloodstream through caries lesions [10] or periodontal pocket of ulcers [11, 12], which trigger the body's inflammatory response or activate specific cytokines [13], so altering the progression of a variety of systemic diseases [9].

A limited number of studies have found a relationship between poor oral health habits and chronic illnesses. Three longitudinal studies showed an inverse association between the frequency of brushing and subsequent cardiovascular events among adults in Japan, China and Korea, respectively [14–16]. Local and systemic inflammation triggered by bacteria associated with periodontal illnesses is hypothesized to contribute to the development of atherosclerosis [17–19], which is linked to an increased risk of cardiovascular diseases (CVD) [20] and pulmonary disease [9].

Meanwhile, previous research has shown an association between poor oral hygiene and an increased risk of developing diabetes [21, 22]. It’s possible that *Porphyromonas gingivalis* (*P. gingivalis*) in periodontal tissues causes insulin resistance and obesity [23], or that it causes the destruction of islet β-cells through an immune inflammatory response, which subsequently results in diabetes [24]. However, another study found no association between the frequency of brushing and diabetes [16]. Additionally, a small number of other studies have discovered a link between poor oral health behavior and an increased risk of liver diseases [16, 25] and upper aerodigestive tract cancer risk [26]. Nevertheless, the existing studies have shown that the strength of the association between oral health practices and chronic conditions may vary across ethnic or geographic populations [19, 27], and a few studies have suggested the degree of the association between brushing practices and various chronic diseases, including musculoskeletal, digestive, respiratory, and genitourinary diseases [25, 28]. Moreover, another study from China [16] measured oral health behavior with regular brushing and never or rarely brushing rather than the number of daily brushing sessions, and its findings were limited in accurately guiding interventions and policy-making.

Therefore, the aim of this study was to evaluate the association between oral health behavior and various chronic diseases among middle-aged and older adults, using a large survey data from Beijing, the capital of China. This study will contribute to revealing more risk factors for chronic diseases and has implications for efforts to oral health in China.

## Methods

### Data source

Data used in this study came from Beijing Health Service Survey, carried out in 2018 by the Beijing Municipal Health Commission. Multistage stratified cluster random sampling was used to select households from 16 districts of Beijing. In the first two stages, probability proportional to size sampling (PPS) was used to respectively select townships (streets) and villages (communities) according to their population size. Then 60 households were selected by simple random sampling from each selected village (community). Each household member was interviewed face-to-face by the investigator using a tablet computer (PAD). All participants gave their informed consent.

The survey tapped a wide range of information for each member of the household, including age, sex, education, income, marital status, living arrangement, region, health behavior, self-reported health status, needs and utilization on health care services, health insurance coverage. Further details of the survey content are accessible on the website http://www.nhc.gov.cn/mohwsbwstjxxzx/dczlxz/201810/ba05fccd129543af912dd0ea447d4d5d.shtml. A total of 12,303 households with 29,202 members of all ages were investigated. Of those, 18,188 adults aged 45 years or older were selected as our interested research population. After excluding 30 respondents with incomplete data, 18,158 middle-aged and older adults were included in the final analysis.

### Measures

#### Outcomes

The outcome of interest was common chronic diseases in China, with chronic disease defined as a disease that is long-lasting with persistent effects [29, 30]. Diseases were selected and grouped according to six major groups of commonly reported diseases in China [29]. Subjects were asked 3 questions, the first two were, “Do you suffer from doctor-diagnosed hypertension (HTN)?”, “Do you suffer from doctor-diagnosed diabetes mellitus (DM)?”, possible options were “Yes” or “No”. The third question was “Do you suffer from doctor-diagnosed other chronic diseases?”, and the respondents could choose up to three other chronic diseases from a list of 132 common chronic diseases from 21 major groups [31]. The top six categories of diseases with the highest prevalence of chronic diseases among Chinese people [29] were chosen as the main dependent variables, which included diseases of the circulatory system (mainly CVD in this study), endocrine or nutritional metabolic diseases, diseases of the musculoskeletal system, digestive system, respiratory system and genitourinary system. Then, we divided these majors into subgroups for further analysis, which can be shown in Additional file 1: Table S1. The outcome variable of interest excluded the “others” group because it contained too many diseases in the major group to be misleading and difficult to interpret.

#### Exposure

Self-reported frequency of daily toothbrushing was used to measure oral health behavior. Subjects were asked “How many times do you brush your teeth on average every day?”, possible answers were 2 or more, once, less than 1, and never. We divided the frequency of toothbrushing into both categorical variables and continuous variables to analyze the differences in risk of chronic diseases among different toothbrushing groups, and to examine whether there was a dose–response relationship between the frequency of brushing and chronic diseases. The two groups of categorical variables of toothbrushing were 2 or more and 1 or less, and the continuous variables were assigned 0, 1, 2 in order according to the frequency of brushing was 0 or < 1, 1, 2 or more.

#### Confounders

Potential confounding variables incorporated into this study were age, gender (male, female), marital status (married, unmarried or other), education (primary school or less, junior high school or high school, college or more), living arrangement (living alone, living with family numbers), household per capita income, region (urban, rural), health insurance (Urban Employee Basic Medical Insurance (UEBMI), Urban and Rural Resident Basic Medical Insurance (URRBMI)), smoking status (yes, no), drinking status (yes, no), physical activity (yes, no), body mass index (BMI, kg/m^2^) (< 18.5, 18.5 ≤ 24.0, 24.0 ≤ 28.0, 28.0 or more).

### Statistical analysis

Participants’ characteristics were analyzed descriptively. Means and standard deviations were used to describe continuous characteristics, and proportions were used for categorical variables. A chi-square test was used to analyze categorical variables. Multivariate logistic models were used to examine the association between oral hygiene behavior and the risk of chronic diseases in various systems. We estimated the association between oral hygiene behavior and the risk of various chronic diseases by fitting logistic regression models, initially evaluated in a crude model and then progressively adjusted for demographic variables, socioeconomic status, and health behaviors or status. In addition, we ran trend tests with frequency of brushing as a continuous variable to assess the robustness of our findings. Odds ratio (OR) with corresponding 95% confidence intervals (CI) were reported. P values less than 0.05 were considered statistically significant. All statistical analyses were conducted in Stata 16.0 (StataCorp LP, College Station, Texas).

## Results

Among 18,158 middle-aged and older adults included, the mean age was 61.34 years (SD 10.26) and 48% were men (Table 1). Overall, 37.70% of participants reported 1 or less brushing their teeth. The prevalence of any chronic diseases was 50.24%, with CVD having the highest prevalence at 40.20%, followed by diseases of the endocrine or nutritional metabolic (15.39%), musculoskeletal (3.79%), digestive (2.04%), respiratory (1.79%) and genitourinary (1.38%) systems. The majority of participants were married, lived with family numbers, attended junior or high school, resided in urban areas, participated in URRBMI, had better lifestyle habits, and were overweight.

**Table 1:** Characteristics of the study population

Characteristics	N (%)
Frequency of toothbrushing	
2 or more	11,313 (62.30)
1 or less	6845 (37.70)
Chronic diseases	9123 (50.24)
Diseases of the circulatory system	7300 (40.20)
Endocrine or nutritional metabolic diseases	2795 (15.39)
Diseases of the musculoskeletal system	689 (3.79)
Diseases of the digestive system	371 (2.04)
Diseases of the respiratory system	325 (1.79)
Diseases of the genitourinary system	250 (1.38)
Age, years, mean (SD)	61.34 (10.26)
Gender	
Male	8778 (48.34)
Female	9380 (51.66)
Marriage status	
Married	16,103 (88.68)
Others	2055 (11.32)
Living arrangement	
Living alone	1938 (10.67)
Living with family numbers	16,220 (89.33)
Education	
Primary or less	3834 (21.11)
Junior or high school	11,745 (64.68)
College or more	2579 (14.21)
Per capital household income (RMB, ten thousands yuan), mean (SD)	3.12 (3.32)
Areas	
Rural	7707 (42.44)
Urban	10,451 (57.56)
Insurance	
UEBMI	7420 (40.86)
URRBMI	10,258 (56.49)
Others	480 (2.64)
Smoke status	
No	13,644 (75.14)
Yes	4514 (24.86)
Drink status	
No	13,490 (74.29)
Yes	4668 (25.71)
Physical activity	
Inactive	4517 (24.88)
Active	13,641 (75.12)
BMI, kg/m^2^	
Underweight	518 (2.85)
Normal weight	7364 (40.56)
Overweight	7570 (41.69)
Obese	2706 (14.90)
Total	18,158

BMI, Body mass index; SD: standard deviation; UEBMI: Urban employee basic medical insurance; URRBMI: Urban and rural resident basic medical insurance

People who brushed their teeth once or less (55.63%) were more likely to have any chronic condition than people who brushed their teeth 2 or more (46.98%), and this difference was statistically significant (*P* < 0.001). Similarly, the prevalence of circulatory, digestive, and genitourinary diseases was 45.96%, 2.09% and 1.61%, respectively, among those who brushed their teeth once a day or less, which was higher than those who brushed twice a day or more (*P* < 0.05), as shown in Table 2.

**Table 2:** The prevalence of different chronic diseases by frequency of tooth brushing

Chronic diseases	Frequency of tooth brushing		*P*-value
	2 or more	1 or less	
Any chronic disease			
Yes	5315 (46.98)	3808 (55.63)	**< 0.001**
No	5998 (53.02)	3037 (44.37)	
Diseases of the circulatory system			**< 0.001**
Yes	4154 (36.72)	3146 (45.96)	
No	7159 (63.28)	3699 (54.04)	
Endocrine or nutritional metabolic diseases			0.754
Yes	1734 (15.33)	1061 (15.50)	
No	9579 (84.67)	5784 (84.50)	
Diseases of the musculoskeletal system			0.271
Yes	443 (3.92)	246 (3.59)	
No	10,870 (96.08)	6599 (96.41)	
Diseases of the digestive system			0.271
Yes	221 (1.95)	150 (2.19)	
No	11,092 (98.05)	6695 (97.81)	
Diseases of the respiratory system			**0.018**
Yes	182 (1.61)	143 (2.09)	
No	11,131 (98.39)	6702 (97.91)	
Diseases of the genitourinary system			**0.038**
Yes	140 (1.24)	110 (1.61)	
No	11,173 (98.76)	6735 (98.39)	

Bold values represents the statistically significant difference in *P* values or 95% CI

In the unadjusted model, people who brushed teeth with 1 or less had a higher risk of any chronic diseases (OR = 1.41, 95% CI: 1.33–1.50), CVD (OR = 1.46, 95% CI: 1.38–1.56), diseases of respiratory (OR = 1.31, 95% CI: 1.05–1.63) and genitourinary (OR = 1.31, 95% CI: 1.02–1.68) system compared with those brushed teeth with 2 or more. After adjusting for demographic factors, socioeconomic status, health behaviors and BMI, the risk of any chronic diseases (OR = 1.27, 95% CI: 1.18–1.37), CVD (OR = 1.30, 95% CI: 1.21–1.39), and endocrine or nutritional metabolic disorders (OR = 1.11, 95% CI: 1.01–1.22) was higher in those who brushed teeth with 1 or less than those who with 2 or more (Table 3).

**Table 3:** Odds ratio (OR) and 95% confidence intervals (95% CI) for the association between frequency of toothbrushing and risk of different chronic diseases

	Model^a^	Model^b^	Model^c^	Model^d^
Any chronic disease				
2 or more	Ref	Ref	Ref	Ref
1 or less	1.41 (1.33, 1.50) ***	1.24 (1.16, 1.32) ***	1.30 (1.21, 1.39) ***	1.27 (1.18, 1.37) ***
Diseases of the circulatory system				
2 or more	Ref	Ref	Ref	Ref
1 or less	1.46 (1.38, 1.56) ***	1.30 (1.22, 1.39) ***	1.32 (1.23, 1.42) ***	1.30 (1.21, 1.39) ***
Endocrine or nutritional metabolic disorders				
2 or more	Ref	Ref	Ref	Ref
1 or less	1.01 (0.93, 1.10)	0.93 (0.86, 1.02)	1.09 (1.01, 1.21)*	1.11 (1.01, 1.22)*
Diseases of the musculoskeletal system				
2 or more	Ref	Ref	Ref	Ref
1 or less	0.91 (0.78, 1.07)	0.87 (0.74, 1.02)	0 .93 (0 .78, 1.11)	0.85 (0.71, 1.02)
Diseases of the digestive system				
2 or more	Ref	Ref	Ref	Ref
1 or less	1.12 (0.91, 1.38)	1.03 (0.83, 1.27)	1.11 (0.88, 1.41)	1.13 (0.89, 1.43)
Diseases of the respiratory system				
2 or more	Ref	Ref	Ref	Ref
1 or less	1.31 (1.05, 1.63)*	1.07 (0.86, 1.34)	1.20 (0.94, 1.54)	1.20 (0.93, 1.55)
Diseases of the genitourinary system				
2 or more	Ref	Ref	Ref	Ref
1 or less	1.31 (1.02, 1.68)*	1.02 (0.79, 1.32)	1.30 (0.98, 1.74)	1.21 (0.92, 1.63)

Bold values represents the statistically significant difference in *P* values or 95% CI

BMI, Body mass index

^a^Unadjusted model

^b^Adjusted for age, gender, marital status, living arrangement

^c^Adjusted for b + education, urban or rural, income, health insurance

^d^Adjusted for c + smoke status, drink status, physical activity, BMI

*P < 0.05, **P < 0.01, ***P < 0.001

Table 4 showed the association between daily brushing frequencies and chronic disease subgroups of each system. Those who brushed their teeth with 1 or less had an increased risk of HTN (OR = 1.24, 95% CI: 1.16–1.34), ischemic heart disease (IHD) (OR = 1.29, 95% CI: 1.08–1.54), cerebrovascular disease (CD) (OR = 2.12, 95% CI: 1.75–2.57) and DM (OR = 1.13, 95% CI: 1.03–1.24), compared those with 2 or more. However, brushing behavior was not associated with the risk of hyperthyroidism. Similar to the main results, we found no significant relationship between toothbrushing frequency and subtypes of diseases of the musculoskeletal, digestive, respiratory, and genitourinary systems.

**Table 4:** The association between frequency of toothbrushing and risk of different chronic diseases by subgroups

Subgroup of diseases	Frequency of daily booth brushing		OR (95% CI)		*P*-value
	≥ 2	≤ 1			
Diseases of the circulatory system					
HTN	3784 (33.45)	2793 (40.80)		**1.24 (1.16, 1.34)**	**< 0.001**
IHD	348 (3.08)	316 (4.62)		**1.29 (1.08, 1.54)**	**0.004**
CD	221 (1.95)	374 (5.46)		**2.12 (1.75, 2.57)**	**< 0.001**
Endocrine or nutritional metabolic diseases					
DM	1618 (14.3)	1025 (14.97)		**1.13 (1.03, 1.24)**	**0.010**
Hyperthyroidism	30 (0.27)	11 (0.16)		0.55 (0.25, 1.19)	0.130
Diseases of the musculoskeletal system					
Rheumatoid arthrosis	66 (0.58)	40 (0.58)		0.93 (0.60, 1.46)	0.781
Intervertebral disc disease	217 (1.92)	114 (1.67)		0.86 (0.67, 1.12)	0.277
Diseases of the digestive system					
Gastrointestinal tract diseases	128 (1.13)	94 (1.37)		1.18 (0.87, 1.6)	0.280
Liver or gallbladder diseases	221 (1.95)	150 (2.19)		1.13 (0.89, 1.43)	0.302
Diseases of the respiratory system					
Chronic pharyngitis or laryngitis	48 (0.42)	18 (0.26)		0.89 (0.49, 1.64)	0.724
Chronic lung disease	136 (1.20)	126 (1.84)		1.29 (0.97, 1.71)	0.072
Diseases of the genitourinary system					
Kidney or urinary diseases	73 (0.65)	44 (0.64)		1.03 (0.67, 1.58)	0.864
Reproductive diseases	67 (0.59)	67 (0.98)		1.36 (0.93, 1.99)	0.108

Bold values represents the statistically significant difference in *P* values or 95% CI

OR, Odds ratio; CI, confidence intervals; BMI, body mass index. CD: Cerebrovascular disease; CVD: Cardiovascular diseases; DM: Diabetes mellitus; HTN: Hypertension; IHD: Ischemic heart disease

^a^OR and 95% CI adjusted for age, gender, marital status, living arrangement, education, urban or rural, income, health insurance, smoke status, drink status, physical activity, BMI

The trend analysis showed that the risk of chronic disease, CVD, HTN, CD, and DM increased with the decrease in brushing frequency, which was consistent with the main results (data not shown). However, the frequency of toothbrushing was not associated with the risk of IHD when we identified the number of brushes as a continuous variable in the adjusted model.

## Discussion

Overall, our study found that poor oral hygiene practices were associated with chronic diseases, CVD, and endocrine or nutritional metabolic disorders among middle-aged and elderly people. Specifically, less frequent toothbrushing (once or less per day) was associated with HTN, IHD, CD and DM. No significant correlation was found between oral health behavior and the diseases of the musculoskeletal, respiratory, digestive, and genitourinary systems.

Our study showed that poor oral health behavior was associated with a higher risk of CVD, which was consistent with the previous studies [14–16, 19, 32]. The present findings were based on data from a large sample of cross-sectional data in Beijing, which represented the association between oral hygiene behaviors and conditions among middle-aged and older adults in the most developed areas of China. A national population-based survey (mean age 50) in Scotland reported that people with poor oral hygiene (never/rarely brushed teeth) had a higher risk of CVD (HR = 1.7, 95% CI: 1.3–2.3) [19]. Another cohort from Korea (mean age 52) suggested that tooth brushing two times, three times or more a day were associated with HRs of 0.87 (95% CI: 0.83–0.91), 0.79 (95% CI: 0.75–0.83) in cardiovascular events compared with tooth brushing once or less a day after a median follow-up of 9.5 years, respectively [15]. Although these two large longitudinal studies defined outcome variables based on physician judgment or discharge diagnosis rather than self-report by participants, it is regrettable that they did not further examine the association between oral health and risk of specific CVD subgroups. A longitudinal study from the China Kadoorie Biobank (CKB) (mean age 51) indicated that participants who rarely or never brushed teeth had adjusted *HR* of 1.12 (95% CI: 1.09–1.15) for major coronary events, with HRs for CD of 1.06 (95% CI: 1.03–1.09), and IHD of 1.00 (95% CI: 0.97–1.04) compared with those who brushed teeth regularly during a median 9.6 years of follow-up [16]. A sufficiently large sample size (*N* = 487,198) allows the CKB study to identify associations between oral hygiene and multiple vascular (myocardial infarction, cardiovascular death, IHD, ischaemic stroke, intracerebral haemorrhage, subarachnoid haemorrhage and pulmonary heart disease) and non-vascular (DM, chronic kidney disease, cancer, COPD and liver cirrhosis) events. It categorized subjects as “rarely or never” and “sometimes or always” according to the frequency of brushing by self-reported. However, the data used in the current study allowed us to identify the brushing frequency as “1 or less” or “2 or more”, which is more feasible in practical life and is in line with the practice recommended (twice-daily tooth brushing) by the World Health Organization(WHO) [33]. Additionally, the classification in this study has many other strengths. First, it can more clearly reflect the dose–response relationship between tooth brushing frequency and associated chronic diseases. However, rarely or never, sometimes or always such responses that rely on subjective judgment may be misclassified. Second, it can provide evidence for the development of health management policies for middle-aged and elderly people, such as providing more specific and accurate recommendations for brushing norms and guidelines. Third, it can provide more precise intervention recommendations for population health management initiatives and chronic disease prevention and control.

The relationship between oral health behaviors and CVD may be explained by several biological mechanisms. For example, many previous studies have confirmed that poor oral hygiene may increase the potential risk of CVD by affecting vascular endothelial function or inflammatory response [19, 34]. Endothelial dysfunction and systemic inflammation play an important role in the pathogenesis of atherosclerosis, which is a major contributor to cardiovascular events [19, 35]. Besides, Poor oral hygiene contributes to the inflammatory burden by increasing concentrations of both C-reactive protein and fibrinogen and leads to increased CVD [19].

In this study, we demonstrated that less frequent toothbrushing (less that once daily) was associated with a higher risk of endocrine or nutritional metabolic diseases when the model was adjusted for demographic variables, socioeconomic status, smoke and drink behavior, physical activity and BMI. There was no association found prior to adjusting the model. This suggess that the association between the two could be mediated by confounding factors. In general, people with lower education and lower household income tend to have poor oral health behavior [36, 37] and a higher risk of metabolic diseases [38, 39]. Therefore, socioeconomic status may be a regulating factor in the association between oral health behavior and endocrine or nutritional metabolic diseases, however it is not possible to state this based on the findings of the present study alone. It is also possible that low brushing frequency is associated only with DM and not with other endocrine disorders, such as hyperthyroidism, which is in line with the previous findings [21, 40–43]. A recent meta-analysis including 20 studies (one cohort study, 14 case–control studies, and 5 cross-section studies) showed that a lower frequency of toothbrushing was associated with an increased risk of DM (OR: 1.32, 95% CI: 1.19–1.47) [40]. Although results were similar to the present study, however, the mate-analysis included only three studies on middle-aged and older Asian population, and most of the studies were focused on adolescents, thus our study can provide more evidence on the association between oral hygiene and DM in middle-aged and older Asian population. Similarly in southwest China, a cohort study (mean age 44) reported that compared with almost no tooth brushing, tooth brushing at least twice a day was associated with a 35% reduction (HR = 0.65, 95% CI: 0.45–0.94) in DM events after a median follow-up of 6.59 years [21]. It is worth noting that there are significant disparities between north and southwest China, not only in economic elements, but also in residents' health practices and health literacy. For example, 62% of the participants with 2 or more toothbrushing in the present study, while only 27% in the previous study in southwest China [21]. These results may indicate that low brushing frequency is associated with a high risk of DM in individuals from different ages and areas of economic level in China. A possible mechanism for the association between brushing frequency and DM is that low frequent brushing causes increased bacteria in the mouth, and infection of the gums and tissues around the teeth by *P. gingivalis* exacerbates insulin resistance and obesity, both of which were major risk factors for diabetes [23].

We did not observe associations between tooth brushing and the risk of diseases of the musculoskeletal, respiratory, digestive, and genitourinary systems and their subgroups. Previous studies have reported associations between tooth brushing and respiratory diseases [16, 44, 45]. With a large sample size and a median follow-up of 9.6 years, the CKB study indicated that participants who rarely or never brushed teeth had an adjusted *HR* of 1.12 (95% CI: 1.05–1.20) for chronic obstructive pulmonary disease (COPD) [16], however, this study defined oral hygiene differently than the present study, as discussed in the previous paragraph. In addition, lower frequency brushing or periodontal disease were associated with a higher risk of respiratory disease and COPD exacerbations in two case–control studies of hospitalized patients [44, 45]. Possible reasons for the inconsistency between the results of these studies and the current study are differences in study design, population, measurement and classification of diseases. A few studies have found that a lower frequency of tooth brushing is related to a higher prevalence of liver diseases such as non-alcoholic fatty liver disease [25] or cirrhosis [46]. For example, a retrospective and single hospital-based study from Japan (mean age 44) showed that the risk of non-alcoholic fatty liver disease decreased significantly with increasing frequency of brushing teeth, with an OR of 1–2 times daily of 0.85 (95% CI: 0.77–0.95) and 3 times daily of 0.74 (95% CI: 0.67–0.82) compared with those who brush their teeth less than once a day [25]. However, this is a single hospital-based study, evaluation by multicenter studies and meta-analyses by different ethnic groups should be considered. We did not observe associations between toothbrushing and the risk of diseases of the upper or lower respiratory, liver or gallbladder. In the present study, we classified self-reported chronic diseases into crude categories and included liver and biliary diseases as a subgroup to ensure sufficient sample size and statistical power, which may partly explain the different findings. Future studies are needed to verify the association between tooth brushing frequency and the risk of respiratory and digestive diseases.

A few epidemiological studies have reported no relationship between oral hygiene and rheumatoid arthritis [28], which is consistent with our findings. In addition, our data allowed us to report that there was no association between oral hygiene practices and lumbar disc herniation or genitourinary diseases, which had not been reported previously. Our study focused on middle-aged and elderly participants and provided new evidence for the association between oral health behavior and different chronic diseases.

There are a number of limitations to our study. Firstly, our design was a cross-sectional study, which did not allow for assessing the temporal relationship between oral health behavior and chronic diseases. Although a statistically significant association between oral health behavior and the risk of CVD and DM is tenable, a well designed prospective cohort study is required to establish a causal association between oral care activities and health outcomes. Secondly, since the frequency of tooth brushing and chronic diseases were measured by self-reported in the present study, the participants’ responses may be affected by recall biases or undiagnosed diseases. In addition, residual confounding of unknown or unmeasured factors was possible, although we carefully adjusted for identified risk factors, demographic characteristics, socioeconomic status, and health behaviors in the final models. Thirdly, our data have a lack of dental examination information, such as gingival bleeding, dental plaque, tooth loss, or periodontal status, which are also important measurements of oral health. Further studies are needed to examine the associations between more detailed information on oral health and the risk of chronic diseases to complement our findings. Finally, our findings should only be generalized to other populations with caution, since our study only targeted the middle-aged and elderly in north China.

## Conclusions

Our study demonstrated that poor oral hygiene practices were associated with higher risk of chronic diseases, CVD and DM in middle-aged and older adults. Our findings further confirmed and strengthened the suggested association between oral health behavior and different chronic diseases. These findings motivate further studies, including interventional trials, to evaluate whether improved oral health behavior may prevent the incidence of chronic diseases, and thus provide more targeted intervention strategies. Attention should be given to oral health behavior of CVD or DM patients in addition to diet and exercise therapy.

## Supplementary Information

**Additional file 1:** Supplementary tables.

## Data Availability

The datasets used and/or analyzed during the current study are available from the corresponding author on reasonable request.

## Abbreviations

95% CI: 95% Confidence intervals
BMI: Body mass index
CD: Cerebrovascular disease
COPD: Chronic obstructive pulmonary disease
CVD: Cardiovascular diseases
DM: Diabetes mellitus
HTN: Hypertension
IHD: Ischemic heart disease
OR: Odds ratio
*P. gingivalis*: *Porphyromonas gingivalis*
SD: Standard deviation
UEBMI: Urban employee basic medical insurance
URRBMI: Urban and rural resident basic medical insurance
WHO: World Health Organization
