# Supplementary material for: Association between oral health behavior and chronic diseases among middle-aged and older adults in Beijing, China
Source: BMC Oral Health. 2023 Feb 14;23:97. doi: 10.1186/s12903-023-02764-y (PMC9926674; doi:10.1186/s12903-023-02764-y)
Supplement: Supplementary file 1 — Additional file 1: Supplementary tables. [file 12903_2023_2764_MOESM1_ESM.docx]

**Supplementary material**

TableS1 Groups of chronic diseases and subgroups

| Groups of chronic diseases | Subgroups |
| --- | --- |
| Diseases of the circulatory system  (cardiovascular diseases, CVD) | hypertension(HTN),  cerebrovascular diseases (CD),  ischaemic heart disease(IHD) (including angina pectoris, myocardial infarction, other ischaemic heart disease),  others (including chronic rheumatic heart disease, pulmonary heart disease, other types of heart disease or circulatory disease) |
| Endocrine or nutritional metabolic diseases | diabetes mellitus (DM),  hyperthyroidism,  others(including deficiency or poor nutrition, rickets, obesity and overnutrition, other endocrine nutrition and metabolic diseases) |
| Diseases of the musculoskeletal system | rheumatoid arthritis,  intervertebral disc disease,  others(including osteomyelitis, other musculoskeletal disorders) |
| Diseases of the digestive system | gastrointestinal tract diseases(including gastroenteritis, peptic ulcer, appendix disease),  liver or gallbladder disease(including chronic hepatitis, liver cirrhosis, gallstones),  others(including other diseases of the abdominal cavity and digestive system) |
| Diseases of respiratory system | chronic pharyngitis or laryngitis,  chronic lung disease(including emphysema, COPD, asthma, pneumonia),  others (including other respiratory diseases) |
| Diseases of the genitourinary system | kidney or urinary diseases (including nephritis and nephropathy, pyelitis, urinary stones) ,  reproductive diseases (including prostatic hyperplasia or inflammation, breast diseases, salpingitis or ovaritis, uterine and vaginal prolapse),  others(including other genitourinary diseases) |
